# Pilot Study for the Assessment of the Best Radiomic Features for Bosniak Cyst Classification Using Phantom and Radiologist Inter-Observer Selection

**DOI:** 10.3390/diagnostics13081384

**Published:** 2023-04-10

**Authors:** María Aymerich, Mercedes Riveira-Martín, Alejandra García-Baizán, Mariña González-Pena, Carmen Sebastià, Antonio López-Medina, Alicia Mesa-Álvarez, Gonzalo Tardágila de la Fuente, Marta Méndez-Castrillón, Andrea Berbel-Rodríguez, Alejandra C. Matos-Ugas, Roberto Berenguer, Sebastià Sabater, Milagros Otero-García

**Affiliations:** 1 Diagnostic Imaging Research Group, Galicia Sur Health Research Institute, Hospital Álvaro Cunqueiro, 36312 Vigo, Spain; alejandra.garcia.baizan@sergas.es (A.G.-B.); marina.gonzalez@iisgaliciasur.es (M.G.-P.); marta.mendez-castrillon.susin@sergas.es (M.M.-C.); andrea.berbel.rodriguez@sergas.es (A.B.-R.); alejandra.cecilia.matos.ugas@sergas.es (A.C.M.-U.); milagros.otero.garcia@sergas.es (M.O.-G.); 2 Medical Physics Research Group, Galicia Sur Health Research Institute, Hospital Álvaro Cunqueiro, 36312 Vigo, Spain; mercedes.riveira@iisgaliciasur.es (M.R.-M.); antonio.lopez.medina@sergas.es (A.L.-M.); 3 Radiology Department, Hospital Álvaro Cunqueiro, 36312 Vigo, Spain; 4 Centre de Diagnòstic per la Imatge Clínic, Hospital Clínic de Barcelona, 08036 Barcelona, Spain; msebasti@clinic.cat; 5 Radiophysics Department, Hospital do Meixoeiro, 36214 Vigo, Spain; 6 Radiology Department, Hospital Universitario Central de Asturias, 33011 Oviedo, Spain; aliciamesaalvarez@gmail.com; 7 Radiology Department, Hospital Povisa, 36211 Vigo, Spain; gtardaguila@povisa.es; 8 Radiation Oncology, Complejo Hospitalario Universitario de Albacete, 02006 Albacete, Spain; roberto0099@yahoo.es (R.B.); ssabaterm@gmail.com (S.S.)

**Keywords:** radiomics, Bosniak cysts, CCR phantom, inter-observer correlation coefficient, classification

## Abstract

Since the Bosniak cysts classification is highly reader-dependent, automated tools based on radiomics could help in the diagnosis of the lesion. This study is an initial step in the search for radiomic features that may be good classifiers of benign–malignant Bosniak cysts in machine learning models. A CCR phantom was used through five CT scanners. Registration was performed with ARIA software, while Quibim Precision was used for feature extraction. R software was used for the statistical analysis. Robust radiomic features based on repeatability and reproducibility criteria were chosen. Excellent correlation criteria between different radiologists during lesion segmentation were imposed. With the selected features, their classification ability in benignity–malignity terms was assessed. From the phantom study, 25.3% of the features were robust. For the study of inter-observer correlation (ICC) in the segmentation of cystic masses, 82 subjects were prospectively selected, finding 48.4% of the features as excellent regarding concordance. Comparing both datasets, 12 features were established as repeatable, reproducible, and useful for the classification of Bosniak cysts and could serve as initial candidates for the elaboration of a classification model. With those features, the Linear Discriminant Analysis model classified the Bosniak cysts in terms of benignity or malignancy with 88.2% accuracy.

## 1. Introduction

Radiomics, a discipline that aroused huge interest in personalized medicine during the past few years, allows the extraction of information from medical images for their further analysis. These mineable data are studied in combination with patient information for clinical decision support [1,2]. Texture analysis allows the quantification of a region of interest by calculating the distribution of voxel gray levels and their relationships, reflecting underlying physiological processes and offering information about the lesion under study.

Different radiomic models in combination with other clinical parameters have been reported for the characterization of renal diseases [3,4,5,6]. The widespread use of medical imaging tests in recent decades led to a significant increase in the incidental detection of renal masses. Most kidney tumors are diagnosed with radiological tests performed for other causes. Hence, imaging plays a key role in the diagnosis and management of these patients. In 1986, Dr. Bosniak established a first classification of cystic renal masses based on CT [7]. This classification, modified in 1996, is now widely accepted and establishes five categories of renal cystic masses, considering types IIF, III and IV as complex cystic lesions [8]. A subclassification of III types into IIIs or IIIn has been proposed, considering that approximately half of the patients with III cysts undergo unnecessary surgeries [9,10].

In the era of personalized management and active surveillance, the differentiation of benign from malignant renal cystic masses is of the utmost importance to establish appropriate patient management [11]. Artificial intelligence tools offer the possibility to develop a useful clinical decision support system based on radiomic features for the characterization of complex cystic renal masses in terms of benignity/malignancy.

Since the diagnosis and management of Bosniak cysts are complex due to the heterogeneity pattern they present in CT images, there is a need to develop diagnostic support tools based on the search for radiomic biomarkers. Works regarding the use of radiomics in cystic lesions have not been extensively studied to date. In recent years, several papers support the hypothesis that the use of quantitative biomarkers could be useful in the classification and prognosis of renal cysts not only to categorize them but also to provide a percentage of potential aggressiveness [12]. Minisk et al. [13] used six first-order features to classify cysts as benign (I and II) or potentially malignant (considered from IIF onwards) from a single slice of the CT scan. The different machine learning models used demonstrated high specificity and low sensitivity. Dana et al. [14] also analyzed the usefulness of machine learning models using radiomic variables to distinguish between benign and malignant renal cysts regardless of their Bosniak class and using pathological or long-term follow-up criteria. Recent works [15,16] address the same problem in both the arterial and nephrographic phases of the CT scan.

Efforts should be made to standardize radiomic studies and to link radiomics features with clinical endpoints so that the results could be validated and implemented in the clinical routine [17]. In this sense, multicenter studies with phantoms are the initial step to study feature robustness in terms of repeatability and reproducibility for dimensionality reduction and feature selection for the subsequent training of a classification model [18]. Moreover, since small variations in segmentations could lead to significant changes in the value of the features, the inter-observer correlations should be calculated when lesions are marked by different radiologists.

A differential aspect of this work compared to other publications on the topic is that it includes a hybrid feature selection approach, using a phantom through the different scanners to analyze the stability and robustness of the variables and transferring these results to clinical cases of Bosniak cysts. This allows understanding in a more detailed way the behavior of the different radiomic features under different conditions (protocol, scanner…) to transfer the problem to Bosniak lesions. Ursprung et al. [17] indicate that one of the initial parameters to take into account in the Radiomics Quality Score (RQS) for radiomic work on renal masses is a phantom study to study feature validity. According to Azadikhan et al. [19], in a meta-analysis of 87 papers, none of them presented the phantom study. This work is focused not only on developing a machine learning model that works as a predictor but also on understanding the role of the features that are used in this pilot study.

This study is a preliminary analysis of the most suitable radiomic variables for the elaboration of classification models of malignancy or benignity of Bosniak cysts. For this purpose, a hybrid approach is performed that reduces the dimensionality based on both the robustness of the variables from several scanners and the correlation in the segmentation in the lesions. Initially, a phantom test–retest, intra-CT and inter-CT feature analysis is presented to select the most robust radiomic features across different machines using a texture phantom on five CT scanners. Then, from the anatomical CT images of the Bosniak cysts, the radiomic variables with the highest inter-observer correlation are selected. Finally, the matching variables between the phantom results and the anatomical images are selected, and their benign/malignant classification capacity is evaluated through machine learning models. The results of this study will help to perform further CT radiomic studies for Bosniak cysts.

## 2. Materials and Methods

### 2.1. Phantom Study

A CCR (Credence Cartridge Radiomics) phantom composed by ten inserts of different materials, such as the one employed by Mackin et al. [20], was selected. Complete images of the phantom were obtained with 5 CT scanners from several vendors and models: one Somaton X.cite (Siemens Healthineers, Erlangen, Germany), two LightSpeed VCT (General Electric Healthcare, Chicago, IL, USA), one Somaton Drive (Siemens Healthineers, Erlangen, Germany) and an Ingenuity (Philips, Amsterdam, The Netherlands). Eight different protocols were applied in each CT scanner. Table 1 summarizes the series acquisition parameters. For simplification purposes, the eight protocols are only detailed in the first scanner. In all protocols, the reconstruction kernel was the default or standard one with different names depending on the manufacturer and machine. The pitch factor was always set to 1, the image matrix was 512 × 512 and the mA value was fixed without any modulation system. FBP corresponds to filtered back projection. The protocols were selected according to previous literature [21]. However, given the intrinsic characteristics of each scanner, some parameters such as FOV, slice thickness or reconstruction algorithm could not be selected exactly the same, although an attempt was made to maximize their similarity in all cases. Tube current modulation was disabled.

Ten regions of interest (ROI) were registered and segmented using ARIA 15.1 software from Varian (Siemens Healthineers, Germany), delimitating a cylindrical ROI with a volume of 116 cm^3^ in each insert of the phantom. This method ensured that the same areas were analyzed in the radiomic study through the different protocols and machines.

Quibim Precision 2.8 platform (Quibim S.L., Valencia, Spain) with CE marking and IBSI compliant was used for the extraction of the radiomic features. Image intensity was normalized and voxels were isotropically resampled to 1 × 1 × 1 mm^3^. The software internally removes the outliers, and it sets distance to neighbor to 1. Shape-related features were not analyzed in this paper, since ROI cylindrical shapes were imposed during registration. The software automatically extracted 105 radiomic features, 14 of them related to shape. Therefore, 91 radiomic features were calculated for each series (see Supplementary Material for the complete list of the radiomic features).

Repeatability and reproducibility assays were performed with the CCR phantom to assess radiomic feature robustness. For test–retest, protocol 1 of each scanner was repeated twice in a five-minute interval without phantom repositioning. Intraclass correlation coefficient (ICC) in two-way and agreement modality [22] was calculated for each pair of acquisitions. Moreover, within-subject coefficient of variation (wCV) was also computed [23]. Finally, the coefficient of variation (CV) as the ratio between standard deviation and the mean value was obtained for each scanner and insert material. R software was employed for the statistical analysis. A feature was considered repeatable when fulfilling ICC ≥ 0.9 and wCV ≤ 1% in, at least, 4 of the 5 CT scanners. Threshold conditions in this work were selected for achieving approximately the same number of radiomic features in each group of analysis.

Reproducibility was divided into intra and inter-CT experiments. In intra-CT studies, the eight protocols were compared pairwise for each scanner. The Concordance Correlation Coefficient (CCC) was calculated for each pair of measurements. Intra-CT reproducibility criteria were CCC ≥ 0.9 and wCV ≤ 10% in 5 or more of the comparisons. From those, only the reproducible features in four CT scanners or more were selected. Inter-CT reproducibility studies compared the feature extraction obtained from different scans using the same acquisition protocol. In this case, CCC and wCV values were calculated. Features with CCC ≥ 0.9 were considered reproducible.

**Figure 1:**
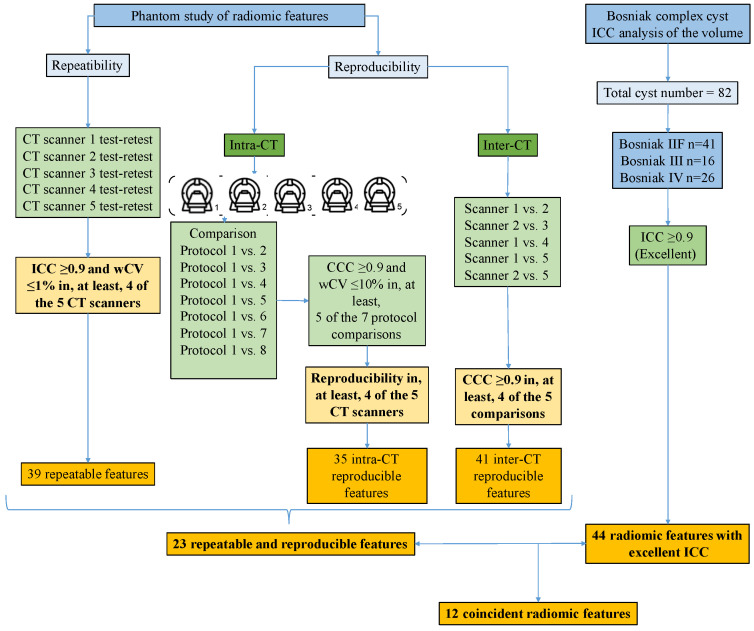
Flowchart of repeatability and reproducibility analysis of phantom assays and its comparison with Bosniak cyst feature statistical analysis.

Finally, features that fulfilled the three criteria of test–retest, intra, and inter-CT were considered robust. A sketch of the experiment is depicted in Figure 1.

### 2.2. Intra-Observer Correlation Coefficient of Radiomic Features in Bosniak Lesions

Subjects were enrolled from June 2019, and 82 cases were selected (mean age 67.86 ± 12.46 years, 51 men). Of these, 41 cysts were diagnosed by expert radiologists in their clinical routine as IIF type, 16 were diagnosed as III type and 26 were diagnosed as IV, following the updated classification proposed in 2019. Inclusion criteria were patients with cystic renal masses who will undergo a CT scan to characterize these masses and who underwent surgery due to the characteristics of the mass, patients with cystic renal masses who underwent biopsy for pathological diagnosis or patients with active surveillance criteria. Exclusion criteria were patients who did not undergo complete multiphase CT due to technical error, patients with renal insufficiency, patients allergic to iodine contrast or patients whose cystic renal masses were not primary. Bosniak cysts IIF that had not changed over the course of two years were considered benign. All Bosniak cyst III and IV included had anatomical pathology confirmation of their benignity or malignancy. The database contains 49 Bosniak cysts considered benign and 33 considered malignant. The demographic information for each group and anatomopathological results from malignant cysts are summarized in Table 2.

In Table 2, qualitative variables were presented as frequency and percentage. For the quantitative variables, the Kolmogorov–Smirnov test was performed to determine if they followed a parametric distribution or not. In the case of age, since it was a parametric variable, it was presented as mean and standard deviation. In cyst size, which follows a non-parametric distribution, the value was presented as median and limit values. For the analysis of statistical significance (considering 0.05 as threshold) in the case of quantitative variables, the chi-square test was used. To compare the means of age according to the benign–malignant group, Student’s t-test was performed. Lastly, to compare the maximum size of the cysts between both groups, as it was a non-parametric variable, the Mann–Whitney U test was performed. No statistically significant differences were found between the two groups. Note that, as reported by Terada et al. [24], men are more likely to suffer from renal cysts than women.

The intravenous contrast phase was selected in each case, as it is the one employed by the clinicians for the classification. Representing a clinical routine sample, series from these subjects were acquired with the same CT scanners employed for the phantom study. This ongoing prospective study received the approval of the Local Ethics Committee, and all participants signed the informed consent.

With the aim of assessing the reliability of radiomic features, the ICC for each feature is calculated by three different radiologists with different degrees of expertise (resident, junior, and senior). They performed the volumetric segmentation of the lesions, and radiomic features were extracted using the same software as in the phantom (Quibim Precision 2.8 platform). ICC among the three radiologists was calculated, and only features with ICC ≥ 0.90 were selected.

### 2.3. Classification Capacity of the Selected Features for Bosniak Cyst Prediction

The selected radiomic features were those resulting from the conducted phantom study and from applying the ICC ≥ 0.90 criteria of the cyst segmentation, obtaining a total of 12 out of 91 features. For comparison purposes with phantom results, shape features were not considered in this study.

To test the ability of the 12 features to distinguish between the benignity and malignancy of cysts included in the database, data were split into a train (65/82) and a test group (17/82), and different machine learning models were trained. The data included in the database were transformed by subtracting the mean and normalizing this value to the unit variance. The pipeline was composed by the following models: Linear Discriminant Analysis (LDA), Support Vector Machine (SVM) and Gaussian Naïve Bayes (GNB) and their corresponding hyperparameters. To optimize the parameters of different models, a grid search space was defined, using the f1-score as the scoring parameter. The cross-validation parameter was set to 10. The performance of each model was evaluated, and the best performing hyperparameter combination was selected.

## 3. Results

### 3.1. Phantom Study

#### 3.1.1. Phantom Repeatability

**Figure 2:**
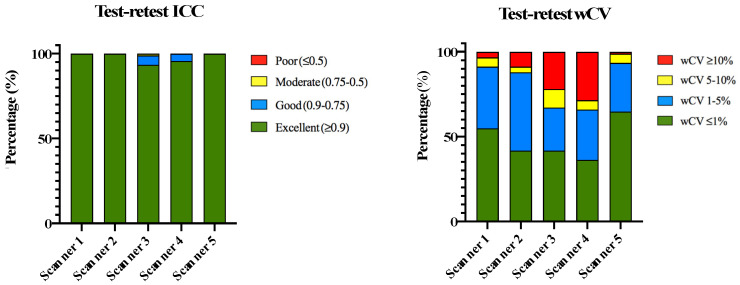
ICC and wCV histograms for the test–retest analysis of the radiomic features for the five CT scanners using protocol 1.

Test–retest was performed on CT series acquired using protocol 1 of each scanner. After rigid registration, segmentation and feature extraction, the radiomic features were obtained. ICC and wCV were calculated for each pair of acquisitions. Results are plotted in Figure 2 and summarized in Table 3.

The ICC value for most of the features was excellent in the five scanners. However, when wCV was calculated, differences among the machines were observed. Scanners 1 and 5 were the most repeatable machines, since all radiomic features presented an excellent ICC, and more than 95% of them had a wCV less than 10%. On the contrary, scanners 3 and 4 were the less repeatable ones. The ICC value was not 100% for all radiomic features in these machines, and the number of features with wCV above 1% was higher for these scanners compared to the others.

Regarding the radiomic variables, features with excellent ICC and wCV *≤* 1% in, at least, four of the five scanners were considered repeatable. This resulted in a set of 39 repeatable features.

For the test–retest experiments, the feature CV for each material and scanner was also calculated. Detailed figures can be found in the Supplementary Material. Regarding the materials, wood was the most repeatable one through different scanners, and polyurethane the least repeatable one.

#### 3.1.2. Phantom Intra-CT Reproducibility

**Figure 3:**
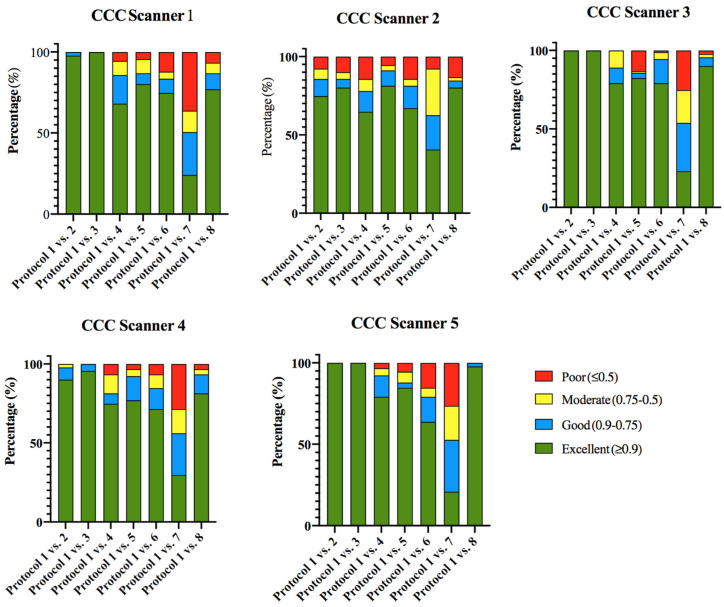
Histograms representing the percentage of features according to their CCC value for the five CT scanners when changing some parameters from protocol 1.

**Figure 4:**
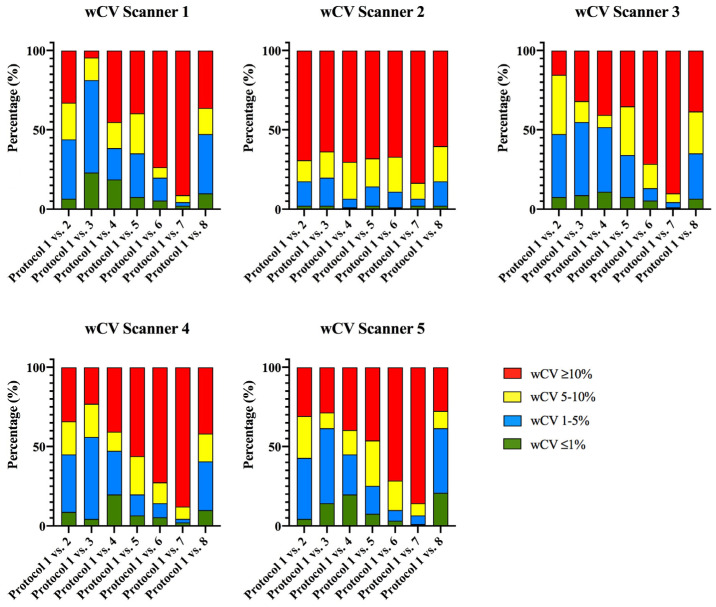
Histograms representing the percentage of features according to their wCV value for the five CT scanners when changing some parameters from protocol 1.

Reproducibility analysis was repeated on the five CT scanners to identify the most reproducible machines and to select the most reproducible features when working with different protocols. Protocol 2 and 3 slightly modified voltage and tube current values. Protocol 4 involved a sharp increase in tube current. In protocols 5 and 6, the slice thickness was halved and doubled, respectively. In protocol 7, the field of view was changed and, finally, in the last protocol, the iterative reconstruction was disabled. CCC and wCV values were calculated, and the results are plotted in Figure 3 and Figure 4. Tables related with these data are available in the Supplementary Material.

In general, a smooth variation of current and voltage conditions (protocol 1 vs. 3 and 1 vs. 2, respectively) had no significant impact on the reproducibility of radiomic features. However, when this change was severe (protocol 1 vs. 4), the number of features with high CCC and low wCV diminished. Slice thickness modification (protocol 1 vs. 5 and 6) was also a source of variation, obtaining higher reproducibility when the thickness was reduced in comparison to when it was increased. The use or lack of iterative reconstruction (protocol 1 vs. 8) also modified the number of reproducible features in a similar way to the tube parameters. The modification of the field-of-view (FOV) (protocol 1 vs. 7) led to the lowest reproducibility of features for all the scanners.

Reproducible features were chosen following the criteria described in the methodology, and the results are presented in Table 4. Siemens Somaton X.cite was the most reproducible scanner regarding parameter modifications (44 of 91 reproducible features) and GE Lightspeed VCT-1 scanner was the least one, with only 24 reproducible variables. A final dataset of intra-CT reproducible features with data from the 5 scanners was composed of 35 out of 91 radiomic features.

In comparison with the test–retest study, the wCV condition was increased from ≤1% to ≤10%, obtaining a final dataset with approximately the same number of features in each section that would allow a final cross-comparison of the results. This relaxation of the constraints is logical, since more feature variation is expected when CT parameters are modified than when the same image is acquired under the same conditions.

#### 3.1.3. Phantom Inter-CT Reproducibility

**Figure 5:**
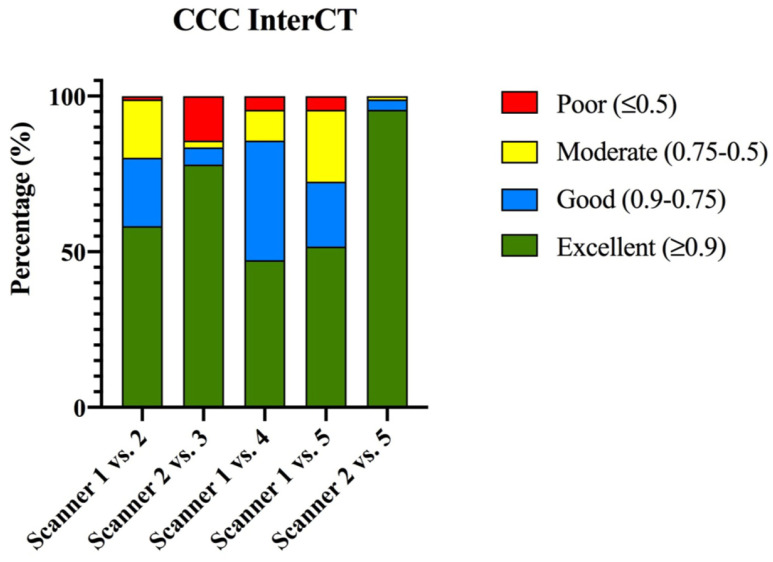
Histogram of the CCC value for the radiomic features when comparing image acquisition with different scanners.

The radiomic feature reproducibility between different scanners was analyzed by calculating the CCC value of the radiomic variables between different scan acquisitions using protocol 1. Results are summarized in Table 4 and depicted in Figure 5.

Figure 5 shows that the most reproducible comparison is between the GE LightSpeed VCT-1 and Philips Ingenuity machines (Scanner 2 vs. 5), where 95.6% of the features presented an excellent CCC. Comparison between the same model and vendor scanners (GE LightSpeed VCT) resulted in a 78.1% of reproducible features. Interestingly, the comparison between both Siemens scanners was the least reproducible, with only 47.2% of the features with an excellent CCC. Nevertheless, even if the vendor was the same for both scanners, the model of the machine was different.

For the selection of the inter-CT variables, 41 out of the 91 features (45.05%) were considered reproducible when working between different CT scanners.

wCV was also computed in this comparison. The results are shown in the Supplementary Material. Considering that in this situation the phantom was not only repositioned but also moved to another machine, the wCV variability of the features was very high in all cases and was not statistically suitable for feature filtering. Nevertheless, a similar tendency to CCC behavior was presented in the inter-CT wCV values. In particular, comparisons between GE LightSpeed VCT scanners and GE LightSpeed VCT-1 and Philips Ingenuity were the most reproducible.

### 3.2. Intra-Observer Correlation Coefficient of Radiomic Features in Bosniak Lesions

From the segmentation by three radiologists with different degrees of experience with Bosniak lesions, it was found that 44/91 variables showed an ICC greater than or equal to 90%, which was considered as excellent. Next, we compared this dataset of features with the one obtained in the previous section, where the most robust features were collected through the different CT scanners. This results in 12 matching features that can be considered candidates for the development of machine learning models for Bosniak cyst classification. Table 5 shows the two lists of variables and, in bold, those that coincide in both datasets.

### 3.3. Classification Capacity of the Selected Features for Bosniak Cyst Prediction

Table 6 shows the results obtained using the variables previously selected with the criteria of the phantom and ICC study. The main classification metrics (accuracy, precision, recall, AUC and f1-score) were calculated. From the numerical data of Table 6, it can be concluded that the model with the best performance is the Linear Discriminant Analysis (LDA), with an accuracy of 88.2%.

**Figure 6:**
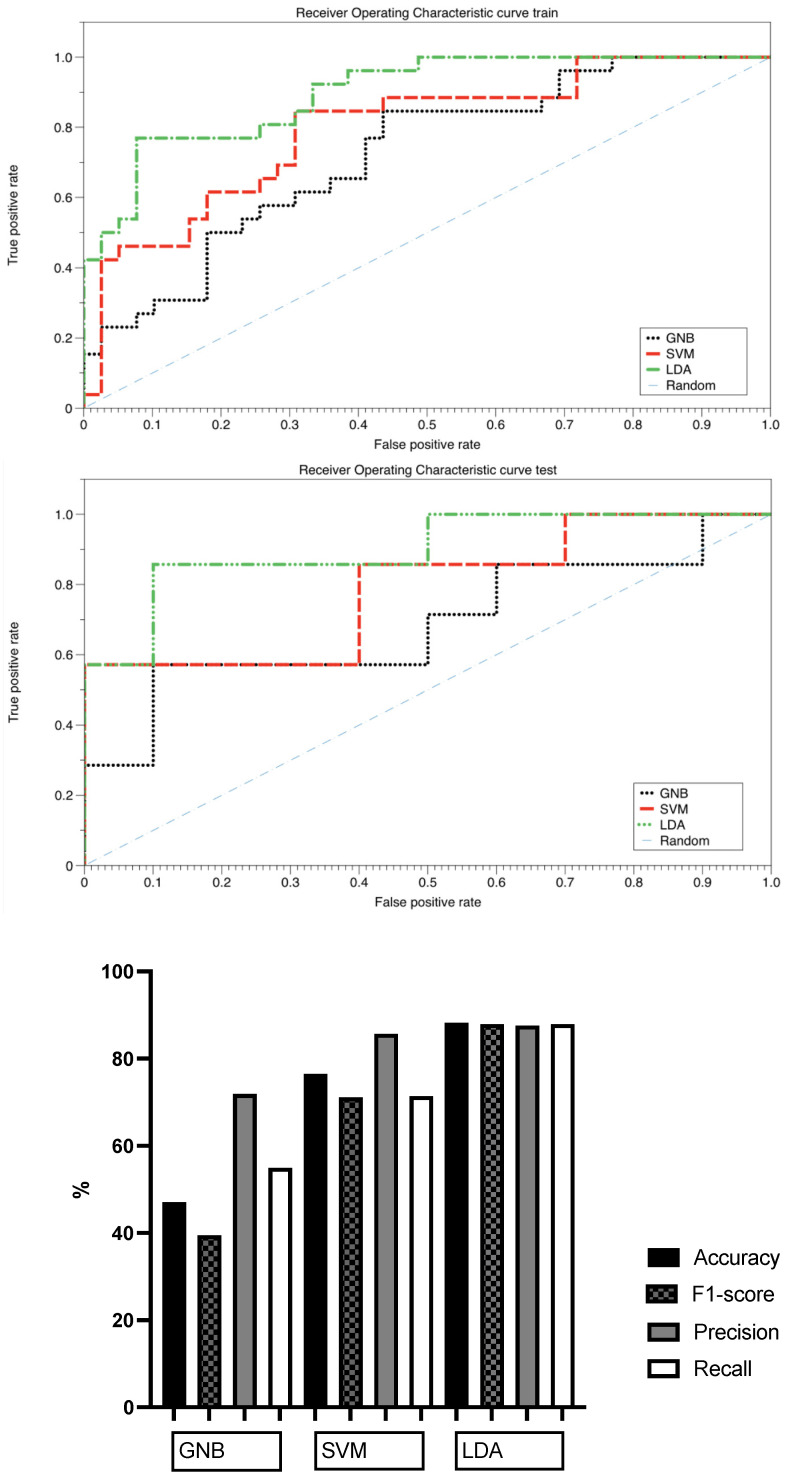
Receiver Operation Characteristic Curve (ROC) for each model and histogram of the obtained metrics.

To evaluate the performance of the classification models, the Receiver Operation Curve (ROC) was calculated. Figure 6 represents the ROC curve from training and test datasets for each of the models included. This curve shows the true positive rate (TPR), represented on the y-axis, and the false positive rate (FPR), represented on the x-axis. Additionally, the area under the curve (AUC) is shown. Figure 6 shows that the most efficient model is the Linear Discriminant Analysis with an AUC = 0.90. Therefore, it can be concluded that the chosen features show good distinguishing ability between malignancy and benignity classes in this model.

## 4. Discussions

In this work, an exhaustive reproducibility and repeatability analysis of feature stability in textural phantoms was presented, obtaining the most robust radiomic features for five CT scanners from different vendors. Overall, 25.3% (23/91) of the studied features were classified as repeatable and reproducible. Moreover, the features that showed an excellent correlation according to the radiologists who segmented the Bosniak lesion (44/91) have been calculated. Features that fulfill both conditions have been selected: stability and robustness through acquisition CT and good inter-observer correlation, obtaining a total number of 12. Finally, the benign/malignant predictive capacity of the Bosniak selected features of cystic renal masses was evaluated, finding that they could be good classifiers for this type of injury. This is an initial study that aims to serve as a basis for the development of future classification models. In particular, entropy-related features have already been shown to be useful in differentiating renal pathologies [17].

Phantom studies have several advantages in comparison with anatomic images, as there is no subject variability [21], and errors in image acquisition due to patient movement are also eliminated. Repositioning in repeatability experiments was avoided to minimize sources of variability [25]. Furthermore, the same feature extraction platform was employed in phantom and cyst experiments, ensuring no possible discordance between feature values related to software design. Studies have demonstrated that the feature calculation platform has a direct impact on radiomics variability, since different types of software return different feature values depending on code design, especially on the utilization of different pre-processing methodologies [26,27].

In the intra-CT reproducibility analysis, it was found that image-related parameters, such as FOV, had a significant impact on feature variability, as has been previously described [28,29], in particular those related to reconstruction more than the acquisition ones [18]. All series were resampled to isotropic voxels before feature calculation, as it was shown as a useful pre-processing technique for feature stability across different protocols [30,31,32], but it seemed clearly ineffective when the FOV was modified. In this work, the modification of the field-of-view value had a direct impact on the robustness of radiomic features, as occurred in other studies [33]. In addition, several CT scanners were used in this work, as multicenter studies allow to increase the statistical power and the reliability of the results, guaranteeing reproducibility across different hospitals [34].

This hybrid approach in the selection of the classifiers, combining the phantom and patient data, allows us to reduce the dimensionality of the model and maintains the features that are robust through the scanners and the segmentation of the lesions of the pathology. Regarding cyst delimitation, one advantage of this work is that a three-dimensional volumetric segmentation of the region of interest was performed. Other works selected only one representative slice of the series for the feature extraction with the subsequent limitations of this approach [5].

The recent version of Bosniak classification included new updates for CT and MRI complex renal cyst diagnosis [35]. Some of these considerations are highly observer-dependent, such as septa and nodule measurement. Image markers such as radiomic features could be helpful in the classification of these complex cysts and could be employed for the development of machine learning models for these purposes. In this work, 12 radiomic features were found as good classifiers for the elaboration of classification models that could predict the benignity or malignity of the lesion [36]. In particular, the LDA model is a good tool for separating groups as in this case. Some sets of features linked to matrices such as NGTDM were discarded as they were neither robust nor informative. Some studies in the literature already support the importance of first-order features as well as entropy characteristics from high-order textures [18], as it was found out in this study for Bosniak cysts.

Although this is a pilot study with a limited number of cases, it is an initial step to determine which radiomic features may be good candidates for the elaboration of a model that serves as a support for the clinical decision on whether the cyst is benign or malignant. As indicated by Krishna et Schieda [12], one of the limitations of the Bosniak 2019 radiological classification is that its final objective is to classify the type of cyst (I-II-IIF-III-IV) and not its malignancy or benignity. Radiomic studies in this direction could be the answer to provide, through imaging, not only the classification but also a prediction of the aggressiveness of the lesion. This would have a direct impact on patient management and better stratification of lesions that are candidates for surgery. A greater number of cases and external validation would allow these results to be brought closer to the clinical routine at a future stage.

This study presents some limitations. Shape features were not analyzed, as cylindrical segmentation was imposed in the phantom acquisitions and there exists inherent feature variability due to the extraction software [37]. In Bosniak cyst analysis, these features were not considered since no comparison with phantom results was possible. Nevertheless, those could be informative for diagnosis purposes, as it was demonstrated in other anatomical areas [38]. Filtering conditions for variable selection were chosen in order to obtain a similar number of robust features in each subset, allowing a final comparison among test–retest, intra-CT and inter-CT stable features. On the other hand, if the filtering conditions were loosened, the number of selected variables would be excessive for the further training of a benignity/malignity diagnosis tool, and a high-dimensionality and redundancy problem will occur [39]. Bosniak cases were not equally selected across the five CT scanners, as they were a representation of clinical routine where most of the renal cyst are discovered incidentally during the image exploration [8]. Nevertheless, benign and malignant cysts were 59.7–40.2%, respectively, of the whole dataset. Moreover, the number of subjects analyzed in this work is limited. A higher number of enrolled participants will be ideal for the next steps in this research. Nevertheless, this study is presented as an initial point for further investigation in Bosniak classification using a radiomics model. Further work related to shape features should be carried out, since some of those features could be useful in the elaboration of an improved model.

There are several initiatives related to the standardization of radiomics pipeline, such as IBSI or EIBALL [40,41]. This study aimed to serve as the starting point for the development of a diagnostic tool based on artificial intelligence that follows the recommended steps to obtain a proper radiomics quality score [17,42]. In this sense, imaging protocols were public, multiple and volumetric segmentations of the lesion were performed, studies with phantoms were carried out to assess the robustness of features, and a dimensionality reduction was proposed when working with renal complex cysts. Overall, 12 features were determined as candidates for classification model training with a special interest in first-order and entropy ones.

## Figures and Tables

**Table 1:** Parameters for the different CT acquisitions. Eight protocols were performed for each of the five scanners.

**CT Scanner 1: Siemens Somaton X.cite**					
	**Tube Voltage** **(kVp)**	**Tube Current** **(mA)**	**Slice Thickness** **(mm)**	**Field of View** **(mm)**	**Reconstruction** **Algorithm**
Protocol 1	120	60	1	600	ADMIRE 3
Protocol 2	100	90	1	600	ADMIRE 3
Protocol 3	140	40	1	600	ADMIRE 3
Protocol 4	120	430	1	600	ADMIRE 3
Protocol 5	120	60	0.5	600	ADMIRE 3
Protocol 6	120	60	2	600	ADMIRE 3
Protocol 7	120	60	1	250	ADMIRE 3
Protocol 8	120	60	1	600	FBP
**CT Scanner 2: GE LightSpeed VCT-1**					
Protocol 1	120	60	1.25	500	ASIR 30%
…	…	…	…	…	…
**CT Scanner 3: GE LightSpeed VCT-2**					
Protocol 1	120	60	1.25	500	ASIR 30%
…	…	…	…	…	…
**CT Scanner 4: Siemens Somaton Drive**					
Protocol 1	120	60	1	500	ADMIRE 3
…	…	…	…	…	…
**CT Scanner 5: Philips Ingenuity**					
Protocol 1	120	60	1	500	iDose 4
…	…	…	…	…	…

**Table 2:** Demographic information of the benign and malignant cysts groups. The anatomopathological results of malignant cysts are presented.

		Benign Cysts (*n* = 49)	Malignant Cysts (*n* = 33)	*p* Value
Sex	Women (*n* = 28)	16 (32.7%)	12 (36.4%)	0.728
	Men (*n* = 51)	33 (67.3%)	21 (63.6%)	
Mean age (years)		67.39 ± 13.47	67.33 ± 11.39	0.985
Cyst location	Left kidney (*n* = 45)	26 (53.1%)	19 (57.6%)	0.687
	Right kidney (*n* = 37)	23 (46.9%)	14 (42.4%)	
Maximum diameter (mm)		33 (12–235)	40 (5–76)	0.653
Anatomopathological result			Clear cell renal cell carcinoma (*n* = 20)	
			Chromophobe renal cell carcinoma (*n* = 5)	
			Papillary type I (*n* = 3)	
			Papillary type II (*n* = 3)	
			Multilocular cyst renal neoplasm of low malignant potential (*n* = 2)	

**Table 3:** Interclass correlation coefficient (ICC) and within-subject coefficient of variation (wCV) values classified for the 91 radiomic features computed. Repeatability analysis was performed over the five CT scanners using protocol 1 of each machine. Test and retest acquisitions were obtained consecutively and without repositioning.

Test–Retest Repeatability Analysis					
**ICC**	**Scanner 1**	**Scanner 2**	**Scanner 3**	**Scanner 4**	**Scanner 5**
Excellent (≥0.9)	100% (91/91)	100% (91/91)	93.4% (85/91)	95.6% (87/91)	100% (91/91)
Good (0.9–0.75)	0% (0/91)	0% (0/91)	5.5% (5/91)	4.4% (4/91)	0% (0/91)
Moderate (0.75–0.5)	0% (0/91)	0% (0/91)	1.1% (1/91)	0% (0/91)	0% (0/91)
Poor (≤0.5)	0% (0/91)	0% (0/91)	0% (0/91)	0% (0/91)	0% (0/91)
**wCV**	**Scanner 1**	**Scanner 2**	**Scanner 3**	**Scanner 4**	**Scanner 5**
≤1%	54.9% (50/91)	41.8% (38/91)	41.8% (38/91)	36.2% (33/91)	64.8% (59/91)
1–5%	36.3% (33/91)	46.1% (42/91)	25.3% (23/91)	29.7% (27/91)	28.6% (26/91)
5–10%	5.5% (5/91)	3.3% (3/91)	10.9% (10/91)	5.5% (5/91)	5.5% (5/91)
≤10%	3.3% (3/91)	8.8% (8/91)	22% (20/91)	28.6% (26/91)	1.1% (1/91)

**Table 4:** Intra and inter-CT reproducibility results of the five different scanners regarding the 91 radiomic features analyzed. In all inter-scanner comparisons, protocol 1 of each CT was employed.

**Intra-CT Reproducibility Analysis**					
	Scanner 1	Scanner 2	Scanner 3	Scanner 4	Scanner 5
**CCC ≥ 0.9 and wCV ≤ 10% in, at least, 5 of the 7 protocol comparisons**	48.4% (44/91)	26.4% (24/91)	46.2% (42/91)	42.9% (39/91)	44% (40/91)
**Inter-CT Reproducibility Analysis**					
**CCC**	Scanner 1 vs. 2	Scanner 2 vs. 3	Scanner 1 vs. 4	Scanner 1 vs. 5	Scanner 2 vs. 5
Excellent (≥0.9)	58.2% (58/91)	78.1% (71/91)	47.2% (43/91)	51.6% (47/91)	95.6% (87/91)
Good (0.9–0.75)	22% (20/91)	5.5% (5/91)	38.5% (35/91)	20.9% (19/91)	3.3% (3/91)
Moderate (0.75–0.5)	18.7% (17/91)	2.2% (2/91)	9.9% (9/91)	23.1% (21/91)	1.1% (1/91)
Poor (≤0.5)	1.1% (1/91)	14.2% (13/91)	4.4% (4/91)	4.4% (4/91)	0% (0/91)

**Table 5:** Repeatable and reproducible radiomic features from the phantom study and features with an Intraclass Correlation Coefficient (ICC) equal to or greater than 0.90%. Coincident features between phantom study and ICC study are bolded.

Repeatable and Reproducible Features from the Phantom Study		Features with ICC ≥ 0.9 from Bosniak Cyst Studies	
**First order**	**10th percentile** **90th percentile** Energy Entropy Mean absolute deviation **Mean** **Median** **Root mean square** Total energy	**First order**	**10th percentile (ICC = 0.95)**
			**90th percentile (ICC = 0.97)**
			Interquartile range (ICC = 0.92)
			Kurtosis (ICC = 0.92)
			**Mean (ICC = 0.96)**
			**Median (ICC = 0.96)**
			Robust mean absolute deviation (ICC = 0.91)
			**Root mean square (ICC = 0.96)**
			Uniformity (ICC = 0.90)
			Major axis length (ICC = 0.92)
			Minor axis length (ICC = 0.97)
			Least axis length (ICC = 0.96)
			Maximum 2D diameter (x-z plane) (ICC = 0.96)
			Maximum 2D diameter (y-z plane) (ICC = 0.90)
			Maximum 3D diameter (ICC = 0.91)
			Area (ICC = 092)
			Voxel volume (ICC = 0.99)
**GLCM**	**Difference average** **Difference entropy** **Joint entropy** Inverse difference moment normalized **Inverse difference** Inverse difference normalized Sum entropy	**GLCM**	Contrast (ICC = 0.94)
			Cluster prominence (ICC = 0.93)
			Cluster tendency (ICC = 0.91)
			Correlation (ICC = 0.90)
			Joint energy (ICC = 0.94)
			Imc1 (ICC = 0.92)
			Imc1 (ICC = 0.94)
			Maximum probability (ICC = 0.96)
			Sum squares (ICC = 0.91)
			**Difference average (ICC = 0.94)**
			**Difference entropy (ICC = 0.93)**
			**Joint entropy (ICC = 0.90)**
			Inverse difference moment (ICC = 0.94)
			**Inverse difference (ICC = 0.94)**
			Inverse variance (ICC = 0.94)
**GLRLM**	Run entropy **Run length non uniformity** **Run length non uniformity normalized** Run percentage **Short run emphasis**	**GLRLM**	Gray-level non-uniformity (ICC = 0.99)
			Long-run emphasis (ICC = 0.96)
			**Run length non-uniformity (ICC = 0.99)**
			**Run length non-uniformity normalized (ICC = 0.90)**
			Run variance (ICC = 0.96)
			**Short-run emphasis (ICC = 0.91)**
**GLSZM**	Zone entropy	**GLSZM**	Large area low gray-level emphasis (ICC = 0.95) Zone variance (ICC = 0.98)
**GLDM**	Dependence entropy	**GLDM**	Dependence non-uniformity (ICC = 0.99) Dependence variance (ICC = 0.96) Gray-level non-uniformity (ICC = 0.99) Large dependence emphasis (ICC = 0.94)

**Table 6:** Classification report for each model of the pipeline. The main metrics are represented: accuracy, f1-score, precision, recall and area under the curve (AUC).

Model	Accuracy	F1-Score	Precision	Recall	AUC
Gaussian Naïve Bayes (GNB)	0.471	0.395	0.719	0.550	0.69
Support Vector Machine (SVM)	0.765	0.717	0.857	0.714	0.79
Linear Discriminant Analysis (LDA)	0.882	0.879	0.876	0.879	0.90

## Data Availability

The data presented in this study are available on request from the corresponding author. The data are not publicly available due to local Ethics Committee decision.

## Supplementary Materials

The following supporting information can be downloaded at: https://www.mdpi.com/article/10.3390/diagnostics13081384/s1.

## References

[1] Rogers W., Seetha S.T., Refaee T.A.G., Lieverse R.I.Y., Granzier R.W.Y., Ibrahim A., Keek S.A., Sanduleanu S., Primakov S.P., Beuque M.P.L. (2020). Radiomics: From qualitative to quantitative imaging. Br. J. Radiol..

[2] Tomaszewski M.R., Gillies R.J. (2021). The Biological Meaning of Radiomic Features. Radiology.

[3] Ding J., Xing Z., Jiang Z., Chen J., Pan L., Qiu J., Xing W. (2018). CT-based radiomic model predicts high grade of clear cell renal cell carcinoma. Eur. J. Radiol..

[4] Lubner M.G. (2020). Radiomics and Artificial Intelligence for Renal Mass Characterization. Radiol. Clin..

[5] Kocak B., Durmaz E.S., Erdim C., Ates E., Kaya O.K., Kilickesmez O. (2020). Radiomics of renal masses: Systematic review of reproduci-bility and validation strategies. AJR Am. J. Roentgenol..

[6] Yu H., Scalera J., Khalid M., Touret A.-S., Bloch B.N., Li B., Qureshi M.M., Soto J.A., Anderson S.W. (2017). Texture analysis as a radiomic marker for differentiating renal tumors. Abdom. Radiol..

[7] Bosniak M.A. (1986). The current radiological approach to renal cysts. Radiology.

[8] Bosniak M.A., Rofsky N.M. (1996). Problems in the detection and characterization of small renal masses. Radiology.

[9] Sefik E., Bozkurt I.H., Adibelli Z.H., Aydin M.E., Celik S., Oguzdogan G.Y., Basmaci I., Gorgel S.N., Vardar E., Gunlusoy B. (2019). The Histopathologic Correlation of Bosniak 3 Cyst Subclassification. Urology.

[10] Pruthi D.K., Liu Q., Kirkpatrick I.D., Gelfond J., Drachenberg D.E. (2018). Long-term surveillance of complex cystic renal masses and het-erogeneity of Bosniak 3 lesions. J. Urol..

[11] Narayanasamy S., Krishna S., Shanbhogue A.P., Flood T.A., Sadoughi N., Sathiadoss P., Schieda N. (2019). Contemporary update on imaging of cystic renal masses with histo-pathological correlation and emphasis on patient management. Clin. Radiol..

[12] Krishna S., Schieda N. (2022). Advances in Imaging of Cystic Renal Masses: Appraisal of Emerging Evidence from Bosniak Version 2019 to Use of Artificial Intelligence. Adv. Clin. Rad..

[13] Miskin N., Qin L., Matalon S.A., Tirumani S.H., Alessandrino F., Silverman S.G., Shinagare A.B. (2020). Stratification of cystic renal masses into benign and potentially malignant: Applying machine learning to the bosniak classification. Abdom. Radiol..

[14] Dana J., Lefebvre T.L., Savadjiev P., Bodard S., Gauvin S., Bhatnagar S.R., Forghani R., Hélénon O., Reinhold C. (2022). Malignancy risk stratification of cystic renal lesions based on a contrast-enhanced CT-based machine learning model and a clinical decision algorithm. Eur. Radiol..

[15] He Q.H., Tan H., Liao F.T., Zheng Y.N., Lv F.J., Jiang Q., Xiao M.Z. (2022). Stratification of malignant renal neoplasms from cystic renal lesions using deep learning and radiomics features based on a stacking ensemble CT machine learning algorithm. Front. Oncol..

[16] He Q.H., Feng J.J., Lv F.J., Jiang Q., Xiao M.Z. (2023). Deep learning and radiomic feature-based blending ensemble classifier for malig-nancy risk prediction in cystic renal lesions. Insights Imaging.

[17] Ursprung S., Beer L., Bruining A., Woitek R., Stewart G.D., Gallagher F.A., Sala E. (2020). Radiomics of computed tomography and magnetic resonance imaging in renal cell carcinoma—A systematic review and meta-analysis. Eur. Radiol..

[18] Traverso A., Wee L., Dekker A., Gillies R. (2018). Repeatability and Reproducibility of Radiomic Features: A Systematic Review. Int. J. Radiat. Oncol..

[19] Azadikhah A., Varghese B.A., Lei X., Martin-King C., Cen S.Y., Duddalwar V.A. (2022). Radiomics quality score in renal masses: A systematic assessment on current literature. Br. J. Rad..

[20] Mackin D., Fave X., Zhang L., Fried D., Yang J., Taylor B., Rodriguez-Rivera E., Dodge C., Jones A.K., Court L. (2015). Measuring CT scanner variability of radiomics features. Investig. Radiol..

[21] Berenguer R., del Rosario Pastor-Juan M., Canales-Vazquez J., Castro-García M., Villas M.V., Masilla Legorburo F., Sabater S. (2018). Radiomics of CT Features May Be Nonreproducible and Redundant: Influence of CT Acquisition Parameters. Radiology.

[22] Koo T.K., Li M.Y. (2016). A Guideline of Selecting and Reporting Intraclass Correlation Coefficients for Reliability Research. J. Chiropr. Med..

[23] Obuchowski N.A., Reeves A.P., Huang E.P., Huang E.P., Wang X.-F., Buckler A., Kim H.J., Barnhart H.X., Jackson E., Giger M. (2015). Quantitative imaging biomarkers: A review of statistical methods for computer algorithm comparisons. Stat. Methods Med. Res..

[24] Terada N., Arai Y., Kinukawa N., Yoshimura K., Terai A. (2004). Risk factors for renal cysts. BJU Int..

[25] Bianchini L., Santinha J., Loução N., Figueiredo M., Botta F., Origgi D., Cremonesi M., Cassano E., Papanikolaou N., Lascialfari A. (2020). A multicenter study on radiomic features from T_2_-weighted images of a customized MR pelvic phantom setting the basis for robust radiomic models in clinics. Magn. Reson. Med..

[26] Fornacon-Wood I., Mistry H., Ackermann C.J., Blackhall F., McPartlin A., Faivre-Finn C., Price G., O’Connor J.P.B. (2020). Reliability and prognostic value of radiomic features are highly dependent on choice of feature extraction platform. Eur. Radiol..

[27] Dreyfuss L.D., Abel E.J., Nystrom J., Stabo N.J., Pickhardt P.J., Lubner M.G. (2021). Comparison of CT Texture Analysis Software Plat-forms in Renal Cell Carcinoma: Reproducibility of Numerical Values and Association With Histologic Subtype Across Platforms. AJR Am. J. Roentgenol..

[28] Midya A., Chakraborty J., Gönen M., Do R.K., Simpson A.L. (2020). Influence of CT acquisition and reconstruction parameters on radiomic feature reproducibility. J. Med. Imaging.

[29] Zhao B. (2021). Understanding Sources of Variation to Improve the Reproducibility of Radiomics. Front. Oncol..

[30] Larue R.T., van Timmeren J.E., de Jong E.E., Feliciani G., Leijenaar R.T.H., Schreurs W.M.J., Sosef M.N., Raat F.H.P.J., Van Der Zande F.H.R., Das M. (2017). Influence of gray level discretization on radiomic feature stability for different CT scanners, tube currents and slice thicknesses: A comprehensive phantom study. Acta Oncol..

[31] Shafiq-Ul-Hassan M., Zhang G.G., Latifi K., Ullah G., Hunt D.C., Balagurunathan Y., Abdalah M.A., Schabath M.B., Goldgof D.G., Mackin D. (2017). Intrinsic dependencies of CT radiomic features on voxel size and number of gray levels. Med. Phys..

[32] Ligero M., Jordi-Ollero O., Bernatowicz K., Garcia-Ruiz A., Delgado-Muñoz E., Leiva D., Mast R., Suarez C., Sala-Llonch R., Calvo N. (2020). Minimizing acquisition-related radiomics variability by image resampling and batch effect correction to allow for large-scale data analysis. Eur. Radiol..

[33] Foy J.J., Al-Hallaq H.A., Grekoski V., Tran T., Guruvadoo K., Iii S.G.A., Sensakovic W.F. (2020). Harmonization of radiomic feature variability resulting from differences in CT image acquisition and reconstruction: Assessment in a cadaveric liver. Phys. Med. Biol..

[34] Kalendralis P., Traverso A., Shi Z., Zhovannik I., Monshouwer R., Starmans M.P.A., Klein S., Pfaehler E., Boellaard R., Dekker A. (2019). Multicenter CT phantoms public dataset for radiomics reproducibility tests. Med. Phys..

[35] Silverman S.G., Pedrosa I., Ellis J.H., Hindman N.M., Schieda N., Smith A.D., Remer E.M., Shinagare A.B., Curci N.E., Raman S.S. (2019). Bosniak Classification of Cystic Renal Masses, Version 2019: An Update Proposal and Needs Assessment. Radiology.

[36] Avanzo M., Wei L., Stancanello J., Vallières M., Rao A., Morin O., Mattonen S.A., El Naqa I. (2020). Machine and deep learning methods for radiomics. Med. Phys..

[37] Doshi A.M., Tong A., Davenport M.S., Khalaf A.M., Mresh R., Rusinek H., Schieda N., Shinagare A.B., Smith A.D., Thornhill R. (2021). Assessment of Renal Cell Carcinoma by Texture Analysis in Clinical Practice: A Six-Site, Six-Platform Analysis of Reliability. Am. J. Roentgenol..

[38] Chaddad A., Desrosiers C., Toews M., Abdulkarim B. (2017). Predicting survival time of lung cancer patients using radiomic analysis. Oncotarget.

[39] Lu L., Ehmke R.C., Schwartz L.H., Zhao B. (2016). Assessing Agreement between Radiomic Features Computed for Multiple CT Imaging Settings. PLoS ONE.

[40] Zwanenburg A., Vallières M., Abdalah M.A., Aerts H.J.W.L., Andrearczyk V., Apte A., Ashrafinia S., Bakas S., Beukinga R.J., Boellaard R. (2021). The Image Biomarker Standardization Initiative: Standardized Quantitative Radiomics for High-Throughput Image-based Phenotyping. Radiology.

[41] Fournier L., Costaridou L., Bidaut L., Michoux N., Lecouvet F.E., de Geus-Oei L.-F., Boellaard R., Oprea-Lager D.E., Obuchowski N.A., Caroli A. (2021). Incorporating radiomics into clinical trials: Expert consensus on considerations for data-driven compared to biologically driven quantitative biomarkers. Eur. Radiol..

[42] Lambin P., Leijenaar R.T.H., Deist T.M., Peerlings J., de Jong E.E.C., van Timmeren J., Sanduleanu S., Larue R.T.H.M., Even A.J.G., Jochems A. (2017). Radiomics: The bridge between medical imaging and personalized medicine. Nat. Rev. Clin. Oncol..

