# Supplementary material for: Pilot Study for the Assessment of the Best Radiomic Features for Bosniak Cyst Classification Using Phantom and Radiologist Inter-Observer Selection
Source: Diagnostics (Basel). 2023 Apr 10;13(8):1384. doi: 10.3390/diagnostics13081384 (PMC10137782; doi:10.3390/diagnostics13081384)
Supplement: Supplementary file 1 [file diagnostics-13-01384-s001.zip › diagnostics-2300321-supplementary.pdf]

Electronic Supplementary Material

Coefficient of variation of each insert material in the test-retest analysis

Coefficient of variation (CV) was calculated for each material in the test–retest analysis to identify for which material the radiomic features were more repeatable. Results are depicted in Figure SM1.

Regarding phantom inserts, as can be seen in Figure SM1, wood is one of the materials that presents a higher percentage of features with a CV <1% across the five CT scanners, and it is always positioned in the first or second place of materials with the lowest coefficient of variation in the test–retest analysis. Concerning the material with higher feature variability, the evidence is not as clear as in the previous case. Polyurethane, for three of the five CT scanners, is the material with the least number of features with CV <1%, suggesting that it is one of the least repeatable.

As it was described in the main text, scanners 1 and 5 presented, in general, a higher number of materials with lower CV (<5%) for most of the materials. This is consistent with the results obtained regarding the ICC and wCV values of the repeatability study.

Within-subject coefficient of variation in inter-CT reproducibility

The tendency of the percentage of reproducible features in comparisons between different CT scanners was similar to the one observed in the CCC analysis, presented in the body of the text, and for the wCV. When comparing scanners 2 and 3 and scanners 2 and 5, the highest number of features with wCV <10% was obtained. On the contrary, and consistent with the CCC results, the inter-CT reproducibility was worse when comparing scanners 1 and 4 and 1 and 5.

However, as already explained, the wCV filter to select the most robust features was not chosen in

this part of the analysis, since it is such a restrictive criterion that it would not allow obtaining a final number of repeatable and reproducible radiomic features comparable with the results obtained in Bosniak cyst study. As an example, if features were filtered with a wCV <10% only 15 reproducible markers would be obtained.

Throughout this work, filtering methods were selected ensuring that each sub-dataset had a comparable number of features for its final comparison. Moreover, it seems intuitive to establish different variability criteria for different tests. In this sense, more restrictive conditions were required in repetition tests where the phantom did not change the machine or protocol and they were relaxed in the case of repositioning and different CT scanners.

Supplementary Material Figure

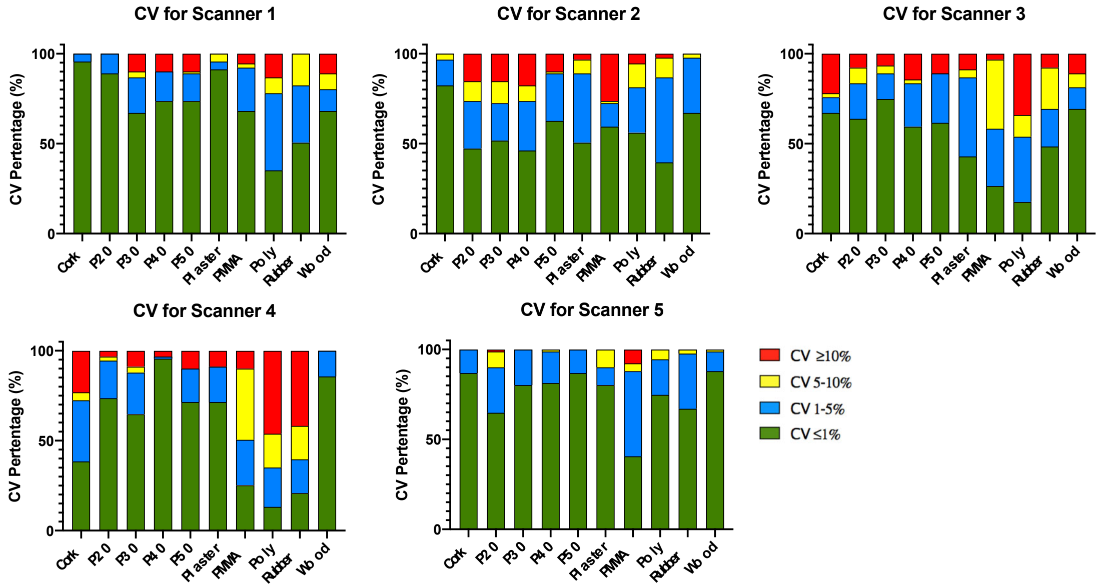

Figure S1: Histograms for the five scanners representing the percentage of radiomic features with different CV values regarding the phantom insert.

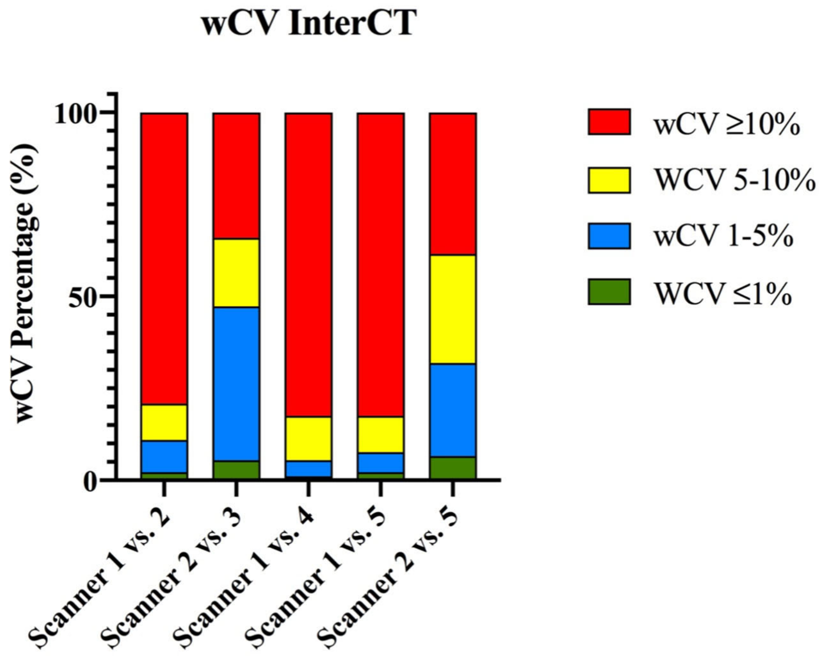

Figure S2: Percentage of radiomic features with different wCV values for different inter-CT comparisons. Protocol 1 of each scanner was employed.

Supplementary Material Tables

Complete list of radiomic features

List of radiomic features	
First Order	10th percentile
	90th percentile
	Energy
	Entropy
	Interquartile range
	Kurtosis
	Maximum
	Mean absolute deviation
	Mean
	Median
	Minimum
	Range
	Robust mean absolute deviation
	Root mean squared
	Skewness
	Total energy
	Uniformity
	Variance
Gray Level Cooccurrence Matrix (GLCM)	Autocorrelation
	Joint average
	Cluster prominence
	Cluster shade
	Cluster tendency
	Contrast
	Correlation
	Difference average
	Difference entropy
	Difference variance
	Joint energy
	Joint entropy
	Informational measure of correlation 1
	Informational measure of correlation 2
	Inverse difference moment
	Inverse difference moment normalized
	Inverse difference
	Inverse difference normalized
	Inverse variance
	Maximum probability
	Sum entropy
	Sum squares
Gray-Level Run Length	Gray-level non-uniformity

Matrix (GLRLM)	Gray-level non-uniformity normalized
	Gray-level variance
	High gray-level run emphasis
	Long-run emphasis
	Long-run high gray-level emphasis
	Long-run low gray-level emphasis
	Low gray-level run emphasis
	Run entropy
	Run length non-uniformity
	Run length non-uniformity normalized
	Run percentage
	Run variance
	Short-run emphasis
	Short-run high gray-level emphasis
	Short-run low gray-level emphasis
Gray-Level Size Zone Matrix (GLSZM)	Gray-level non-uniformity
	Gray-level non-uniformity normalized
	Gray-level variance
	High gray-level zone emphasis
	Large area emphasis
	Large area high gray-level emphasis
	Large area low gray-level emphasis
	Low gray-level zone emphasis
	Size zone non-uniformity
	Size zone non-uniformity normalized
	Small area emphasis
	Small area high gray-level emphasis
	Small area low gray-level emphasis
	Zone entropy
	Zone percentage
	Zone variance
Gray-Level Dependence Matrix (GLDM)	Dependence entropy
	Dependence non-uniformity
	Dependence non-uniformity normalized
	Dependence variance
	Gray-level non-uniformity
	Gray-level variance
	High gray-level emphasis
	Large dependence emphasis
	Large dependence high gray-level emphasis
	Large dependence low gray-level emphasis
	Low gray-level emphasis
	Small dependence emphasis
	Small dependence high gray-level emphasis
	Small dependence low gray-level emphasis
Neighboring Gray Tone	Busyness

Difference Matrix (NGTDM)	Coarseness
	Complexity
	Contrast
	Strength

Table S1: Complete list of the radiomic features that were extracted with Quibim Precision software. Shape features were not taken into account in this work, since regions of interest were predefined.

CCC and wCV tables for intra-CT reproducibility analysis

CT Scanner 1							
**CCC**	*Protocol 1 vs. 2*	*Protocol 1 vs. 3*	*Protocol 1 vs. 4*	*Protocol 1 vs. 5*	*Protocol 1 vs. 6*	*Protocol 1 vs. 7*	*Protocol 1 vs. 8*
Excellent (≥0.9)	97.8% (89/91)	100% (91/91)	68.1% (62/91)	80.2% (73/91)	74.7% (68/91)	24.2% (22/91)	76.9% (70/91)
Good (0.9-0.75)	2.2% (2/91)	0% (0/91)	17.6% (16/91)	6.6% (6/91)	8.8% (8/91)	26.4% (24/91)	9.9 % (9/91)
Moderate (0.75-0.5)	0% (0/91)	0% (0/91)	8.8% (8/91)1	8.8% (8/91)	4.4% (4/91)	13.2% (12/91)	6.6 % (6/91)
Poor (≤0.5)	0% (0/91)	0% (0/91)	5.5% (5/91)	4.4% (4/91)	12.1% (11/91)	36.2% (33/91)	6.6% (6/91)
**wCV**	*Protocol 1 vs. 2*	*Protocol 1 vs. 3*	*Protocol 1 vs. 4*	*Protocol 1 vs. 5*	*Protocol 1 vs. 6*	*Protocol 1 vs. 7*	*Protocol 1 vs. 8*
≤1%	6.6% (6/91)	23.1% (21/91)	18.7% (17/91)	7.7% (7/91)	5.5% (5/91)	2.2% (2/91)	9.9% (9/91)
1-5%	37.4% (34/91)	58.2% (53/91)	19.8% (18/91)	27.5% (25/91)	14.3% (13/91)	2.2% (2/91)	37.4% (34/91)
5-10%	23.1% (21/91)	14.3% (13/91)	16.5% (15/91)	25.3% (23/91)	6.6% (6/91)	4.4% (4/91)	16.5% (15/91)
≥10%	32.9% (30/91)	4.4% (0/91)	45.0% (41/91)	39.6% (36/91)	73.6% (67/91)	91.2% (83/91)	36.2% (33/91)

CT Scanner 2							
**CCC**	*Protocol 1 vs. 2*	*Protocol 1 vs. 3*	*Protocol 1 vs. 4*	*Protocol 1 vs. 5*	*Protocol 1 vs. 6*	*Protocol 1 vs. 7*	*Protocol 1 vs. 8*
Excellent (≥0.9)	74.8% (68/91)	80.2% (73/91)	64.8% (59/91)	81.3% (74/91)	67.0% (61/91)	40.7% (37/91)	80.2% (73/91)
Good (0.9-0.75)	10.9% (10/91)	5.5% (5/91)	13.2% (12/91)	9.9% (9/91)	14.3% (13/91)	21.9% (20/91)	4.4% (4/91)
Moderate (0.75-0.5)	6.6% (6/91)	4.4% (4/91)	7.7% (7/91)	3.3% (3/91)	4.4% (4/91)	29.7% (27/91)	2.2% (2/91)
Poor (≤0.5)	7.7% (7/91)	9.9% (9/91)	14.3% (13/91)	5.5% (5/91)	14.3% (13/91)	7.7% (7/91)	13.2% (12/91)
**wCV**	*Protocol 1 vs. 2*	*Protocol 1 vs. 3*	*Protocol 1 vs. 4*	*Protocol 1 vs. 5*	*Protocol 1 vs. 6*	*Protocol 1 vs. 7*	*Protocol 1 vs. 8*
≤1%	2.2% (2/91)	2.2% (2/91)	1.1% (1/91)	2.2% (2/91)	1.1% (1/91)	2.2% (2/91)	2.2% (2/91)
1-5%	15.4% (14/91)	17.6% (16/91)	5.5% (5/91)	12.1% (11/91)	9.9% (9/91)	4.4% (4/91)	15.4% (14/91)
5-10%	13.2% (12/91)	16.5% (15/91)	23.1% (21/91)	17.6% (16/91)	22.0% (20/91)	9.9% (9/91)	22.0% (20/91)
≥10%	69.2% (63/91)	63.7% (58/91)	70.3% (69/91)	68.1% (62/91)	67.0% (61/91)	83.5% (76/91)	60.4% (55/91)

CT Scanner 3							
**CCC**	*Protocol 1 vs. 2*	*Protocol 1 vs. 3*	*Protocol 1 vs. 4*	*Protocol 1 vs. 5*	*Protocol 1 vs. 6*	*Protocol 1 vs. 7*	*Protocol 1 vs. 8*
Excellent (≥0.9)	100% (91/91)	100% (0/91)	79.2% (72/91)	82.4% (75/91)	79.2% (72/91)	23.1% (21/91)	90.1% (82/91)
Good (0.9-0.75)	0% (0/91)	0% (0/91)	9.9% (9/91)	3.3% (3/91)	15.3% (14/91)	30.7% (28/91)	5.5% (5/91)

Moderate (0.75-05)	0% (0/91)	0% (0/91)	10.9% (10/91)	1.1% (1/91)	4.4% (4/91)	20.9% (19/91)	2.2% (2/91)
Poor (≤0.5)	0% (0/91)	0% (0/91)	0% (0/91)	13.2% (12/91)	1.1% (1/91)	25.3% (23/91)	2.2% (2/91)
**wCV**	*Protocol 1 vs. 2*	*Protocol 1 vs. 3*	*Protocol 1 vs. 4*	*Protocol 1 vs. 5*	*Protocol 1 vs. 6*	*Protocol 1 vs. 7*	*Protocol 1 vs. 8*
≤1%	7.7% (7/91)	8.8% (8/91)	10.9% (10/91)	7.7% (7/91)	5.5% (5/91)	1.1% (1/91)	6.6% (6/91)
1-5%	39.5% (36/91)	46.1% (42/91)	40.7% (37/91)	26.4% (24/91)	7.7% (7/91)	3.3% (3/91)	28.5% (26/91)
5-10%	37.4% (34/91)	13.2% (12/91)	7.7% (7/91)	30.7% (28/91)	15.4% (14/91)	5.5% (5/91)	26.4% (24/91)
≥10%	15.4% (14/91)	31.9% (29/91)	40.7% (37/91)	35.2% (32/91)	71.4% (65/91)	90.1% (82/91)	38.5% (35/91)

CT Scanner 4							
**CCC**	*Protocol 1 vs. 2*	*Protocol 1 vs. 3*	*Protocol 1 vs. 4*	*Protocol 1 vs. 5*	*Protocol 1 vs. 6*	*Protocol 1 vs. 7*	*Protocol 1 vs. 8*
Excellent (≥0.9)	90.1% (82/91)	95.6% (87/91)	74.7% (68/91)	76.9% (70/91)	71.4% (65/91)	29.7% (27/91)	81.3% (74/91)
Good (0.9-075)	7.7% (7/91)	4.4% (4/91)	6.6% (6/91)	15.4% (14/91)	13.2% (12/91)	26.4% (24/91)	12.1% (11/91)
Moderate (0.75-0.5)	2.2% (2/91)	0% (0/91)	12.1% (11/91)	4.4% (4/91)	8.8% (8/91)	15.4% (14/91)	3.3% (3/91)
Poor (≤0.5)	0% (0/91)	0% (0/91)	6.6% (6/91)	3.3% (3/91)	6.6% (6/91)	28.5% (26/91)	3.3% (3/91)
**wCV**	Protocol 1 vs. 2	Protocol 1 vs. 3	Protocol 1 vs. 4	Protocol 1 vs. 5	Protocol 1 vs. 6	Protocol 1 vs. 7	Protocol 1 vs. 8
≤1%	8.8% (8/91)	4.4% (4/91)	19.8% (18/91)	6.6% (6/91)	5.5% (5/91)	2.2% (2/91)	9.9% (9/91)
1-5%	36.2% (33/91)	51.6% (47/91)	27.5% (25/91)	13.2% (12/91)	8.8% (8/91)	2.2% (2/91)	30.8% (28/91)
5-10%	20.9% (19/91)	20.9% (19/91)	12.1% (11/91)	24.2% (22/91)	13.2% (12/91)	7.7% (7/91)	17.6% (16/91)
≥10%	34.1% (31/91)	23.1% (21/91)	40.6% (37/91)	56.0% (51/91)	72.5% (66/91)	87.9% (80/91)	41.7% (38/91)

CT Scanner 5							
**CCC**	Protocol 1 vs. 2	Protocol 1 vs. 3	Protocol 1 vs. 4	Protocol 1 vs. 5	Protocol 1 vs. 6	Protocol 1 vs. 7	Protocol 1 vs. 8
Excellent (≥0.9%)	100% (91/91)	100% (91/91)	79.1% (72/91)	84.6% (77/91)	63.7% (58/91)	20.9% (19/91)	97.8% (89/91)
Good (0.9-0.75%)	0% (0/91)	0% (0/91)	13.2% (12/91)	3.3% (3/91)	15.4% (14/91)	31.9% (29/91)	2.2% (2/91)
Moderate (0.75-0.5 %)	0% (0/91)	0% (0/91)	4.4% (4/91)	6.6% (6/91)	5.5% (5/91)	20.9% (19/91)	0% (0/91)
Poor (≤0.5%)	0% (0/91)	0% (0/91)	3.3% (3/91)	5.5% (5/91)	15.4% (14/91)	26.3% (24/91)	0% (0/91)
**wCV**	*Protocol 1 vs. 2*	*Protocol 1 vs. 3*	*Protocol 1 vs. 4*	*Protocol 1 vs. 5*	*Protocol 1 vs. 6*	*Protocol 1 vs. 7*	*Protocol 1 vs. 8*
≤1%	4.4% (4/91)	14.3% (13/91)	19.8% (18/91)	7.7% (7/91)	3.3% (3/91)	1.1% (1/91)	20.9% (19/91)
1-5%	38.5% (35/91)	47.2% (43/91)	25.3% (23/91)	17.6% (16/91)	6.6% (6/91)	5.5% (5/91)	40.7% (37/91)

5-10%	26.4% (24/91)	9.9% (9/91)	15.4% (14/91)	28.6% (26/91)	18.7% (17/91)	7.7% (7/91)	10.9% (10/91)
≥10%	30.7% (28/91)s	28.6% (26/91)	39.5% (36/91)	46.1% (42/91)	71.4% (65/91)	85.7% (78/91)	27.5% (25/91)

Table S2: Concordance correlation coefficient (CCC) and within-subject coefficient of variation (wCV) for the five CT scanners in the intra-CT analysis. The percentage was calculated for the 91 radiomic features studied in this work.

Inter-CT reproducibility analysis					
**wCV**	*Scanner 1 vs. 2*	*Scanner 2 vs. 3*	*Scanner 1 vs. 4*	*Scanner 1 vs. 5*	*Scanner 2 vs. 5*
≤1%	2.2% (2/91)	5.5% (5/91)	1.1% (1/91)	2.2% (2/91)	6.6% (6/91)
1-5%	8.8% (8/91)	41.8% (38/91)	4.4% (4/91)	5.5% (5/91)	25.2% (23/91)
5-10%	9.9% (8/91)	18.7% (17/91)	12.1% (11/91)	9.9% (9/91)	29.8% (27/91)
≥10%	79.1% (72/91)	34.0% (31/91)	82.4% (75/91)	82.4% (75/91)	38.4% (35/91)

Table S3: Within-subject coefficient of variation (wCV) for the inter-CT analysis. The percentage was calculated for the 91 radiomic features studied in this work.

	**Test–retest**<br><br>*ICC≥0.9 and wCV≤1% in, at least, 4 of the 5 CT scanners*	**Intra-CT**<br><br>*CCC ≥0.9 and wCV ≤10% in, at least, 5 of the 7 protocol comparisons*	**Inter-CT**<br><br>*CCC ≥0.9*
First Order	10th percentile<br>90th percentile<br>Energy<br>Entropy<br>Interquartile range<br>Mean absolute deviation<br>Mean<br>Median<br>Robust mean absolute deviation<br>Root mean squared<br>Total energy<br>Variance	10th percentile<br>90th percentile<br>Energy<br>Entropy<br>Interquartile range<br>Maximum<br>Mean absolute deviation<br>Mean<br>Median<br>Range<br>Robust mean absolute deviation<br>Root mean squared<br>Total energy<br>Variance	10th percentile<br>90th percentile<br>Energy<br>Entropy<br>Mean absolute deviation<br>Mean<br>Median<br>Minimum<br>Root mean squared<br>Total energy<br>Uniformity
Gray-Level Co-occurrence Matrix (GLCM)	Cluster prominence<br>Cluster shade<br>Cluster tendency<br>Contrast<br>Difference average<br>Difference entropy	Joint average<br>Cluster tendency<br>Difference average<br>Difference entropy<br>Joint entropy<br>Inverse difference	Joint average<br>Difference average<br>Difference entropy<br>Joint entropy<br>Inverse difference moment<br>Inverse difference

	Difference variance<br>Joint entropy<br>Inverse difference moment normalized<br>Inverse difference<br>Inverse difference normalized<br>Sum entropy<br>Sum squares	moment normalized<br>Inverse difference<br>Inverse difference normalized<br>Sum entropy<br>Sum squares	moment normalized<br>Inverse difference<br>Inverse difference normalized<br>Sum entropy
Gray-Level Run Length Matrix (GLRLM)	Gray-level variance<br>Long-run emphasis<br>Run entropy<br>Run length non-uniformity<br>Run length non-uniformity normalized<br>Run percentage<br>Short run emphasis	Gray-level variance<br>Run entropy<br>Run length non-uniformity<br>Run length non-uniformity normalized<br>Run percentage<br>Short run emphasis	Gray-level non-uniformity<br>Gray-level non-uniformity normalized<br>Low gray-level run emphasis<br>Run entropy<br>Run length non-uniformity<br>Run length non-uniformity normalized<br>Run percentage<br>Short run emphasis<br>Short run low gray-level emphasis
Gray-Level Size Zone Matrix (GLSZM)	Gray-level variance<br>Size zone non-	Size zone non-uniformity	Size zone non-uniformity

	uniformity<br><br>Zone entropy<br><br>Zone percentage	normalized<br><br>Small area emphasis<br><br>Zone entropy	Size zone non-uniformity normalized<br><br>Small area emphasis<br><br>Zone entropy<br><br>Zone percentage
Gray-Level Dependence Matrix (GLDM)	Dependence entropy<br><br>Gray-level variance<br><br>Small dependence emphasis	Dependence entropy<br><br>Gray-level variance	Dependence entropy<br><br>Gray-level non-uniformity<br><br>Low gray-level emphasis<br><br>Small dependence emphasis<br><br>Small dependence low gray-level emphasis
Neighboring Gray Tone Difference Matrix (NGTDM)			Coarseness<br><br>Contrast

Table S4: Complete list of radiomic features that fulfill each of the criteria for the test–retest analysis, intra-CT and inter-CT. Only the features that meet the three conditions will be considered stable and robust.
